# Development of a Dew/Frost Point Temperature Sensor Based on Tunable Diode Laser Absorption Spectroscopy and Its Application in a Cryogenic Wind Tunnel

**DOI:** 10.3390/s18082704

**Published:** 2018-08-17

**Authors:** Wei Nie, Zhenyu Xu, Ruifeng Kan, Jun Ruan, Lu Yao, Bin Wang, Yabai He

**Affiliations:** 1 Key Laboratory of Environmental Optics and Technology, AnHui Institute of Optics and Fine Mechanics, Chinese Academy of Sciences, Hefei 230031, China; wnie@aiofm.ac.cn (W.N.); zyxu@aiofm.ac.cn (Z.X.); ruanjun@aiofm.ac.cn (J.R.); lyao@aiofm.ac.cn (L.Y.); yabaihe@hotmail.com (Y.H.); 2 University of Science and Technology of China, Hefei 230031, China; 3 China Aerodynamics Research and Development Center, Mianyang 621000, China; nudtwangbin@163.com

**Keywords:** dew/frost point temperature, TDLAS, saturated vapor pressure, wind tunnel

## Abstract

We have proposed a sensor for real-time and online measurement of dew/frost point temperature using tunable diode laser absorption spectroscopy (TDLAS) technique. Initial experiments have demonstrated its feasibility and technical advantages in comparison to a chilled mirror hygrometer (CMH). The TDLAS sensor we developed has a dew/frost point temperature range from −93 °C to + 14.5 °C, with a measurement uncertainly of less than 2%, and a response time of about 0.8 s, which is much faster than that of the chilled mirror hygrometer (ranging from several minutes to several hours). A TDLAS-based dew/frost point sensor has many advantages, such as rapid and continuous measurements, low frost point temperature sensing, high accuracy, and non-intrusiveness. Such a sensor would be useful for dew/frost point temperature determinations in various applications. In a cryogenic wind tunnel, real-time dew/frost point temperature measurements are helpful in preventing the formation of condensed liquid and ice, which can affect the model geometry and lead to unreliable test data.

## 1. Introduction

Dew point and frost point temperatures are important parameters in meteorological, aerospace, industrial, petrochemical, and other fields. Dew/frost point temperature (commonly referred to as ‘dew/frost point’) is the temperature at which water vapor in any gas medium at a constant pressure begins to condense into liquid water or solid ice at the same rate at which it evaporates, which is a measure of how much water vapor is in a gas medium. For gas temperatures above 0 °C, water vapor condenses as liquid water (dew). A liquid condensation layer is considered a dew point. For gas temperatures far below 0 °C, water vapor condenses as solid ice (frost). A solid condensation layer is considered a frost point. However, for gas temperatures between 0 °C and approximately −20 °C, the state of the condensed layer is undetermined; it may be either water or ice, or some combination of the two. In a cryogenic wind tunnel, accurate and rapid determination of the dew/frost temperature is of great importance, because it shows the amount of water vapor within the tunnel circuit. More importantly, real-time online dew/frost point measurements can not only prevent the wind tunnel water vapor frosting from affecting the experimental results, but can also prevent exposed experimental models from being damaged by the dew/ice in the high-speed flow field [1,2,3].

Various types of humidity sensors have served in dew/frost point measurements. These include aluminum oxide sensors, capacitive thin-film polymer sensors [4], electronic humid sensors [5,6], nanopore sensors [7], polymer thin films [8], quartz crystal resonant (QCR) sensors [9], silicon-only capacitive dewpoint sensors [10], and more. Generally, these sensors are not suitable for harsh environments (caustic, high temperature and low temperature) and are slow to respond (in very low dew point conditions). Chilled mirror hygrometry (CMH) [11] has usually been used to measure dew point in cryogenic wind tunnels. Its operation principle is reliable and mature but the mirror surface can be easily affected by pollutants and cold water, which might lead to inaccurate measurement results. Furthermore, the response speed of the chilled-mirror method is very slow for extremely low frost point temperature measurements (e.g., below −70 °C); it can range from about 30 min to even several hours. Reactive gases such as chlorine, sulfur oxides, and ammonia tend to gradually react with the water dew deposit on the mirror surface, forming an acid or caustic substance that causes incorrect (i.e., higher than normal) equilibrium dew point readings. The CMH method becomes unsuitable in such cases. The classification of some commercially available dew-point instruments according to brand and sensor type are listed in Table 1.

Optical measurement techniques, such as tunable diode laser absorption spectroscopy (TDLAS) are well-established techniques for concentration measurements of gas molecules. Buchholz et al. used TDLAS to achieve airborne humidity measurements, and presented a laboratory-based inter-comparison of a 1.4-μm-diode-laser-based optical hygrometer with the two most important measurement principles for airborne hygrometry (frost-point hygrometers, FPH, and Lyman-alpha fluorescence hygrometers, LAFH). They achieved excellent agreement with reference sensors [12]. In 2017, they designed a dual near-infrared wavelength airborne humidity sensor. This humidity sensor used a direct tunable diode laser absorption spectroscopy (dTDLAS) approach to cover a large atmospheric humidity measurement range from about 3 to 40,000 ppmv, with a calculated maximum uncertainty of 4.3% ± 3 ppmv [13]. Thornberry et al. developed a hygrometer with a dual-channel, closed-path, tunable diode laser absorption spectrometer for measurements of water vapor concentration and total water in the upper troposphere and low stratosphere. The instrument utilized wavelength-modulated spectroscopy near 2694 nm to achieve high in-flight precision, i.e., better than 0.17 ppm [14]. Dew/frost point measurements based on TDLAS have many advantages, such as simple structure, fast response, high accuracy, and non-intrusiveness; also, they are calibration-free and easily miniaturized [15]. The TDLAS is highly suitable for real-time measurements and engineering application in various environments [13,14,15,16,17].

In view of the difficulties that exist when using traditional sensors and the CHM technique to measure dew/frost point at cryogenic environments, we have developed a TDLAS-based optical system for dew/frost point temperature measurements. In the present study, we demonstrate the feasibility and advantages of using absorption spectroscopy with a distributed-feedback (DFB) laser at a wavelength of ~ 1.38 μm to determine water vapor dew/frost point temperatures in a cryogenic wind tunnel. The dew/frost point measurement principles and background absorption removing tactics, as well as the procedure and results of the comparison test and validation of the water vapor adsorption and desorption by a gas tube will be described.

## 2. Principles of optical spectroscopy method

A monochromatic light beam passing through a uniform gas medium will lose intensity due to optical absorption by gas molecules. This process can be described by Beer-Lambert law, given by Equation (1).

(1) $$I_{t}=I_{0}\cdot \exp(-k_{\nu }\cdot L)=I_{0}\cdot \exp\left\{-\sum_{j}S_{j}(T)\cdot p\cdot x_{i}\cdot L\cdot \phi_{j}(\nu ,T,p,x_{i})\right\}$$

Here, $k_{\nu }$ is the absorption coefficient, $I_{0}$ and $I_{t}$ are the incident and transmitted light intensities at optical frequency ν, $S_{j}$ is the line strength of transition j, p(Pa) is the gas total pressure, $x_{i}$ stands for the molar fraction of gas spice i, *L*(cm) denotes the effective optical path length, $\phi_{j}$ is the line-shape function of transition j. The absorbance is calculated as $\alpha_{v}=k_{v}\cdot L=\ln(I_{0}/I_{t})$.

The line strength $S_{j}\left(T\right)$ (${\mathrm{cm}}^{-2}\cdot {\mathrm{atm}}^{-1}$) characterizes the strength of a transition j, which is temperature dependent, and can calculate from the partition function, the lower-state energy of the transition, and the value $S_{j}\left(T_{0}\right)$ at reference temperature:

(2) $$S_{j}(T)=S_{j}\left(T_{0}\right)\frac{Q\left(T_{0}\right)}{Q\left(T\right)}\left(\frac{T_{0}}{T}\right)\exp\left[-\frac{hc{E^{\prime \prime }}_{j}}{k}\left(\frac{1}{T}-\frac{1}{T_{0}}\right)\right]\times \left[1-\exp(-\frac{hc\nu_{j0}}{kT})\right]{\left[1-\exp(-\frac{hc\nu_{j0}}{kT_{0}})\right]}^{-1}$$

where Q is the partition function, ${E^{\prime \prime }}_{j}$ (cm^−1^) is the low-state energy. Spectroscopic databases such as HITRAN [18] and HITEMP (for high temperature) [19] have listed some of these parameters. $T_{0}$(K) is a reference temperature (e.g., 296 K), h(J⋅s) is the Planck’s constant, c(cm/s) is the speed of light, k(J/K) is the Boltzmann’s constant.

The most commonly used line-shape function under conventional temperature and pressure conditions is the Voigt profile [17], which is the convolution of a Gaussian function and a Lorentzian function, and is given by Equation (3).

(3) $$\phi_{j}(\nu )=\frac{a}{\pi }\int_{-\infty }^{+\infty }\frac{\exp(-y^{2})}{a^{2}+{\left(w-y\right)}^{2}}dy$$

Here, $a=p\cdot \gamma /\Delta \nu_{D}{}^{\prime }$, $w=\left(\nu -\nu_{0}{}^{\prime }\right)/\Delta \nu_{D}{}^{\prime }$, $\Delta v_{D}{}^{\prime }$ is the Doppler half-width at half-maximum (HWHM),$\nu_{0}{}^{\prime }$(cm^−1^) is the pressure-shifted line-center, γ(cm^−1^.atm^−1^) is the collisional-broadening coefficient of the transition, and p(Pa) is the pressure. A calculation method for Voigt profiles can be taken from the paper by Schreier [20].

The Beer-Lambert law in Equation (1) can be rewritten by integrating absorbance $\alpha_{\nu }$ over the laser frequency ν. Since the line-shape function $\phi_{j}$ is normalized to have unity, the integrated absorbance can be expressed as Equation (4).

(4) $$A=\int_{-\infty }^{+\infty }\alpha_{\nu }d\nu =-\int_{-\infty }^{+\infty }\ln(\frac{I_{t}(\nu )}{I_{0}(\nu )})d\nu =p\cdot x_{i}\cdot L\cdot S(T)$$

where ($p\cdot x_{i}$)(Pa) corresponds the partial pressure e(T) of the gas of interest. The integrated absorbance A can be retrieved from experimental spectral measurements by a Voigt profile fitting procedure. Then the partial pressure e(T) of water vapor can be calculated by Equation (5) while the environmental temperature is known.

(5) $$e\left(T\right)=p\cdot x_{H_{2}O}=\frac{A}{S\left(T\right)\cdot L}$$

Because the maximum partial pressure of water vapor, also known as saturation vapor pressure, is strictly a function of temperature, the dew/frost point temperature is equal to the gas temperature when gas reaches saturation state. Therefore, a determination of the dew/frost point temperature of H_2_O vapor can be achieved by a direct measurement of the partial pressure of water vapor.

The relation between saturated H_2_O vapor pressure and dew/frost point temperature in the pure water vapor system can be described by Murphy and Koop formula [21], and is given by Equations (6) and (7).

For dew point temperature:

(6) $$\begin{array}{l} \ln e_{w}\left(T_{d}\right)=54.842663-6763.22/T_{d}-4.210\ln(T_{d})+0.000367T_{d}+\tanh\{0.0415(T_{d}-218.8)\}\\ \;\;\;\;\times (53.878-1331.22/T_{d}-9.44523\ln(T_{d})+0.014025T_{d}) \end{array}$$

For frost point temperature:

(7) $$\ln e_{w}\left(T_{f}\right)=9.550426-5723.265/T_{f}+3.53068\ln(T_{f})-0.00728332T_{f}$$

where $T_{d}$(K) and $T_{f}$(K) are dew point and frost point temperature. $e_{w}\left(T_{d}\right)$(Pa) and $e_{w}\left(T_{f}\right)$(Pa) are maximum water vapor pressure at the saturator temperature $T_{d}$ and $T_{f}$, respectively. These two formulas are valid only in the pure water vapor system. Humidified air, however, is a multi-component system so that an enhancement factor $f_{w}(p_{s},T_{s})$ for $e_{w}\left(T_{s}\right)$ is needed [22]. The resulting water vapor partial pressure after the expansion is calculated with the Equation (8).

(8) $$e_{w}\left(T_{s}\right)=\frac{e\left(T\right)\cdot p_{s}}{f_{w}\left(p_{s},T_{s}\right)\cdot p_{t}}$$

where e(T) is the water vapor partial pressure in multi-component gas as measured by the TDLAS technology, $e_{w}\left(T_{s}\right)$ is the pure water vapor pressure, $f_{w}(p_{s},T_{s})$ is the enhancement factor, i.e., the correction of non-ideal behavior of water vapor-air mixture at saturator pressure $p_{s}$ and saturator temperature $T_{s}$. $p_{t}$ is the total pressure of test chamber.

As the total chamber pressure increases, the dew point temperature will rise, and gradually approach saturation. Conversely, expanding a compressed gas to atmospheric pressure decreases the partial pressure of all of the component gases, including water vapor, and therefore decreases the dew point temperature of the gas. The term “dew point” is encountered when measuring the dew point temperature of gases at atmospheric pressure. The partial pressure of water vapor, e, in actual pressure should be expanded or lessened in proportion according to Dalton’s law ($P_{1}/P_{2}=e_{1}/e_{2}$), and then calculated dew or frost point by Equations (6)–(8).

## 3. Absorption line selection

The TDLAS sensor has been designed mainly to measure dew/frost point temperature of a cryogenic wind tunnel, and H_2_O vapor was, therefore, the target species. By spectral simulation using line parameters from HITRAN 2012 database, a line pair around 1.38 μm, as shown in Table 2, was selected. This line pair can be covered by one diode laser, and allows us to simultaneously retrieve gas temperature and flow velocity of hypersonic flow field, which has been used in velocity measurements of a combustion heating based hypersonic tunnel [23].

**Figure 1:**
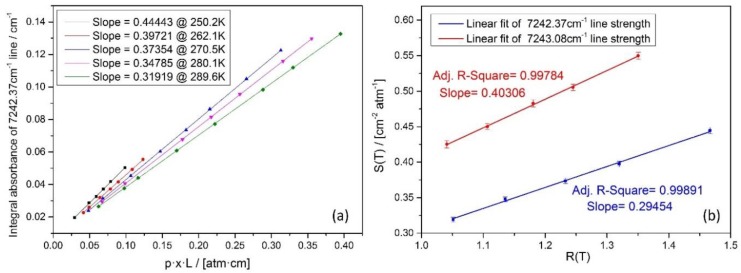
(**a**) Measured integral absorbance of the 7242.37 cm^−1^ line at different pressure and the results of straight line fitting. The linear slope value is the absorption line strength at the corresponding gas temperature. (**b**) straight line fit of the line strength as a function of *R(T)* at different gas temperatures. The linear slope value is the line strength at the reference temperature 296 K.

To achieve accurate water vapor partial pressure determinations by optical absorption spectroscopy, the knowledge of the target absorption line strengths are of primary importance, in addition to the gas total pressure and optical absorption pathlength. For small molecules such as the intensively studied H_2_O, it is possible to estimate its absorption line strengths by using an available spectral database [19] and Equation (2). However, such theoretical calculations might not be accurate enough without further verifications. Therefore, we made calibration measurements for the strengths of the used water vapor absorption lines around 1.38 μm by tunable diode laser absorption spectroscopy in controlled low temperature absorption cell environment in our laboratory. The absorption cell was refrigerated by liquid helium. Its leakage rate was 10^−9^ Pa·m^3^/s, as measured by a helium mass spectrometry leak detector. The accuracy of temperature was less than ± 0.2 K. Silicon bond temperature sensor (DT-670B-CU, Lake Shore Cryotronics Inc., Columbus, OH. USA, temperature tolerance at 100 K–305 K was 0.14%); temperature tolerance at 100 K–305 K was 0.14%) was used to measure water vapor temperature. In addition, the capacitive vacuum gauge (CCR361, Pfeiffer Vacuum Technologies Inc., Wetzlar, Germany, accuracy was 0.2% of the reading) was applied to measure the pressure in the cryogenic absorption cell. Pure water vapor was from a pure water vapor generating device. Before the experiment, the absorption cell was cleaned for several hours by flowing pure water vapor and vacuuming. Then, the absorption spectrum of pure water vapor under different pressures was measured. In the end, the integral absorbances at different pressures were obtained by fitting with a Voigt profile model. Line strength values at each and different measurement temperature were acquired by straight line fitting as illustrated in Figure 1a.

According to the Equation (2), we know that line strength at the actual temperature is in proportion to that at the reference temperature when the low state energy, the partition function value, the gas of temperature, and the frequency of the absorption lines are known. Therefore, the line strength can be rewritten as Equation (9).

(9) S(T)=S(296K)⋅R(T)

where S(296K) is the line strength at a nominal reference temperature $T_{0}=296K$. R(T) represents the rest terms of the right-hand side of Equation (2), which values were based on the HITRAN database.

Measured values of S(T) as a function of calculated values of R(T) were plotted in Figure 1b for both water vapor absorption lines. The linear slope value corresponds to the absorption line strength value at the reference temperature 296 K.

Uncertainty of the obtained line strengths was determined by analyzing the uncertainty of each parameter in Equation (4). Uncertainty of the pressure P and temperature T was 0.2% and 0.14%, respectively. Three main factors influence the uncertainty of the line area: uncertainty in the determination of the optical zero, uncertainty of the fitting and uncertainty of the wavelength/frequency tuning coefficient of the laser. Taking into account these three factors, the uncertainty of integral absorbance of these two lines are about 0.101% and 0.112%. The water reservoir was filled with double distilled water, the purity of which might be affected by gasses dissolved in it during storage. The solubility of common atmospheric gases in water is in the 10 ppm range, which means a relative uncertainty in the water concentration in the 10^−5^ range. This value was used as the uncertainty of $x_{0}$ (the value of $x_{0}$, i.e. the expected amount fraction of H_2_O is 1). During an evacuation of the gas cell, a residual pressure of ~10 Pa was reached, after which the cell was filled with water vapor up to its vapor pressure at room temperature (i.e. ~ 5034 Pa at ~ 33 °C). The maximum error introduced by imperfect evacuation of the gas cell can be calculated as 10/5034 = 0.199%. The uncertainty of effective optical pathlength was estimated to be about 0.20%. In the end, we calculated the uncertainty of line strength at reference temperature based on Equation (10). Our measurement results (Table 2) have improved the accuracy of the values listed in the HITRAN database.

(10) $$\sigma_{S\left(T\right)}=\sqrt{{\left(\frac{\partial S\left(T\right)}{\partial A}\right)}^{2}\sigma_{A}^{2}+{\left(\frac{\partial S\left(T\right)}{\partial p}\right)}^{2}\sigma_{P}^{2}+{\left(\frac{\partial S\left(T\right)}{\partial x}\right)}^{2}\sigma_{x}^{2}+{\left(\frac{\partial S\left(T\right)}{\partial L}\right)}^{2}\sigma_{L}^{2}}$$

where $\sigma_{A}$, $\sigma_{P}$, $\sigma_{x}$, $\sigma_{L}$ is standard deviation of A, p, x, L respectively.

## 4. Experimental setup and data retrieve method

### 4.1. Experimental setup

**Figure 2:**
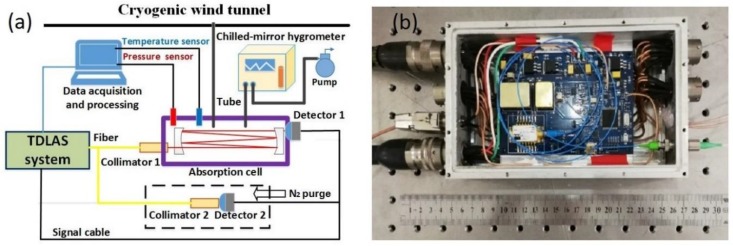
A TDLAS-based sensor for real-time online measurements of dew/frost point temperature in a cryogenic wind tunnel. A chilled-mirror hygrometer was used temporally for comparison measurements. (**a**) The schematic diagram of the experimental setup; (**b**) Photo of the compact TDLAS sub system.

The experimental system developed for dew/frost point temperature measurements in cryogenic wind tunnel is shown in Figure 2. It is based on optical measurements of water vapor partial pressure by using a tunable diode laser absorption spectroscopy (TDLAS) technique. For comparison measurements, a chilled mirror hygrometer is also included in the setup.

The miniaturized TDLAS system (Figure 2b) had dimensions 30 (L) * 14 (W) * 7 (H) cm^3^, and consists of a diode laser source, laser driver (temperature & current control modules), a linear current/frequency scanning circuit, photodetector circuits, and more [12]. One DFB (distributed-feedback) InGaAsP diode laser (nanoplus Nanosystems and Technologies GmbH, Würzburg, Germany) emitting near 1.38 μm was used in this study. The output from the laser was collimated using a parabolic mirror, which is free from any etalon interference a lense might cause. A commercial laser control module (containing a temperature control and current control module) was used to drive the diode laser. A sawtooth signal was utilized to scan the laser injection current, and thus tune the laser wavelengths over the desired absorption features of H_2_O molecules. Transimpedance amplifier circuits were used to convert photocurrent signals to voltage signals. A multipass absorption cell was used to enhance the optical pathlength of 1150.2 cm for low frost-point temperature determination. The absorption cell temperature and pressure were measured with a platinum resistance sensor (accuracy of 0.1% FS) and a pressure sensor (STS Sensor Technik Sirnach AG., Zurich, Switzerland, full-scale 0.5 MPa and accuracy of < 0.05% FS), respectively.

Output beam of the DFB diode laser was divided into two parts by a 1 × 2 fiber splitter. One beam (50%) passed through the 1150.2 cm multipass absorption cell, and was used to measure the optical absorption by water vapor, whereas the intensity of the second beam (50%) was measured by the Detector 2. Both the Detector 2 and the associated Collimator 2 were enclosed in a box which was purged with high purity nitrogen gases to avoid interference from absorption by ambient water vapor. The Detector 2 signal was used to provide background absorption information for correcting the calculation of water vapor absorption spectra measured by Detector 1. The air in the wind tunnel was first introduced into the multipass absorption cell, and then entered the chilled-mirror hygrometer by an air pump. The laser wavelength and the absorption spectra of H_2_O lines were scanned at 5 kHz rate, with photodiode signals sampled at 30 MSam/s. Experimental data acquisition and processing were implemented on LABVIEW software platform. A 1000-scan average was used to improve the signal-to-noise ratio (SNR). The laser absorption Detector 1 signal, the absorption-free Detector 2 signal, and the multipass cell’s temperature and pressure were continuously recorded by a data acquisition card (NI, PXI-5105) installed in a PC for real-time and online processing of data.

### 4.2. The optical pathlength of multipass absorption cell

**Figure 3:**
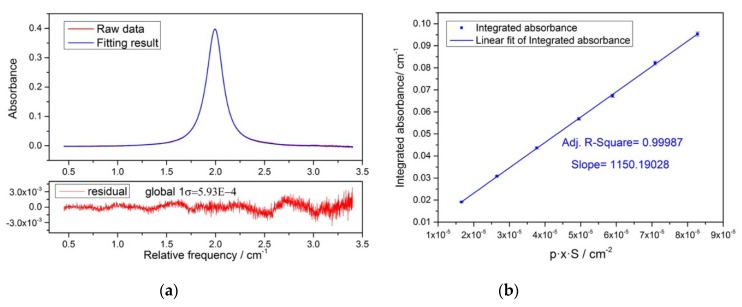
(**a**) Measured methane spectra together with the fitted absorption profile (blue line) and fit residuals, Global 1σ indicates the standard deviation of the residuals in the whole spectral window. (**b**) Straight line fit to the integrated absorption. The slope of the fitting line is the optical path length of the multipass absorption cell.

The multipass absorption cell had a compact, rigid, miniature White type design. The mirror surface diameter was about 7 cm, and the length of the cell was about 50 cm. Sometimes we could estimate the optical pathlength by numbers of light spots and cell length, but this does not meet the requirements for accurate measurement of dew/frost point temperature. We obtained the optical pathlength of multipass absorption cell by making a TDLAS measurement of methane gas with the cell. Because the concentration of methane in the atmosphere is less than 10 ppm [24], we chose a well-characterized methane absorption line around 1.654 μm to prevent interference from gas impurities in the air. The concentration of standard methane gas in this experiment is 1007 ppm in N_2_. We cleaned the absorption cell by the 1007 ppm CH_4_, and by vacuuming for about 30 min, and then let the standard methane gas to fill the absorption cell up to atmospheric pressure. By gradually reducing the total pressure of the absorption cell in steps, we recorded 50 methane gas spectra at each pressure step. Integral absorbance was determined by Voigt profile fitting each spectrum individually and averaging the obtained integral absorbance for the whole measurement sequence. The optical pathlength was obtained by linear fitting the mean of integral absorbance, as shown in Figure 3b. The slope of the fitting line was the desired optical pathlength, i.e., 1150.19 cm.

### 4.3. Data processing and evaluation

**Figure 4:**
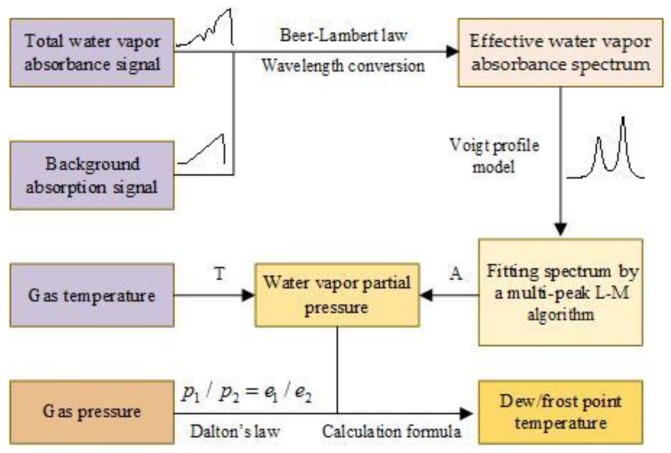
A diagram of the data processing

The data processing of dew/frost point temperature measurement by TDLAS technology was as illustrated in Figure 4.

**Figure 5:**
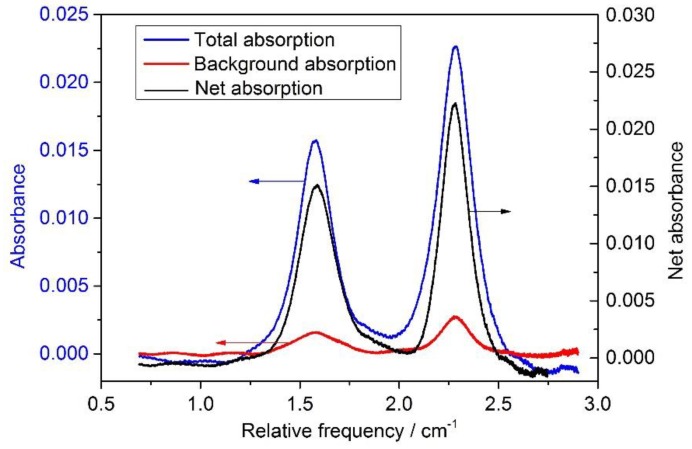
Total absorption (blue line) and background absorption (red line) at a temperature and a pressure. The net absorption (black line) was obtained as their difference.

Background correction was very important for an accurate determination of frost point temperature. For example, a 1-mm long 2%-water-vapor air section along the laser beam path could lead to a ~50% error when the dew point temperature was at about −50 °C. Background absorption could come from the laser package, fiber and connectors, and photodetectors. We calculated the background absorption ($\alpha_{\mathrm{background}}$) reference transmitted signals 2, and then removed $\alpha_{\mathrm{background}}$ from the actual water vapor absorbance ($\alpha_{\mathrm{measurement}}$) signal to obtain the desired net absorption spectrum ($\alpha_{\mathrm{actual}}$) as illustrated in Figure 5.

**Figure 6:**
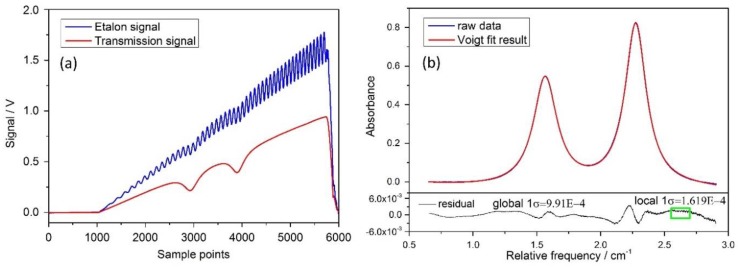
(**a**) A measurement example of etalon interference signal and water vapor absorption signal; (**b**) Water vapor absorption spectrum and multi-peak Voigt profile fit and fitting residual. Global 1σ indicates the standard deviation of the residuals in the whole spectral window, while local 1σ corresponds to the standard deviation of the residuals within the rectangle.

The temporal absorption-free intensity profile $I_{0}(t)$ varied approximately linearly as laser drive current was scanned. We selected the parts of absorption-free intensity profile from the transmission signal $I_{t}(t)$, and then least-square-fitted a third-order polynomial to the absorption-free intensity profile to represent $I_{0}(t)$. It was used late in the calculation of the water vapor absorption according to Beer-Lambert law. The laser wavelength/frequency changes during its drive current scan was predetermined based on interference fringes of a solid etalon with a known free spectral range (FSR), as illustrated in Figure 6a. The absorption spectrum of water vapor was calculated as $\alpha_{v}=\ln(I_{0}/I_{t})$, shown in Figure 6b. Such a measurement absorbance profile covered two absorption lines, and was modeled to a double Voigt profiles to yields the integrated absorbance areas for each transition, as also showed in the Figure 6b. Residuals of those two spectra show higher deviations around the absorption lines, which is a typical indication of jitter, i.e. small fluctuations in the laser current. Line strength was calculated the according to the gas temperature, and water vapor partial pressure according to Equation (5). The gas pressure should be expanded or lessened in proportion to atmospheric pressure, and the partial pressure of water vapor, e, in actual pressure should be expanded or lessened in proportion according to Dalton’s law ($P_{1}/P_{2}=e_{1}/e_{2}$). In the end, the corresponding dew/frost point temperature were then calculated by Equations (6)–(8).

### 4.4. Uncertainty of the sensor

According to the principle of conservation of mass, the mole fraction of water vapor in the saturator $x_{s}$, is equal to that in the test chamber $x_{c}$. Thus

(11) $$x_{s}=x_{c}=f\left(p_{s},T_{s}\right)\cdot e_{w}\left(T_{s}\right)/p_{s}=f\left(p_{c},T_{d/f}\right)\cdot e_{w}\left(T_{d/f}\right)/p_{c}$$

where $f\left(p_{s},T_{s}\right)$ is the enhancement factor at saturation pressure $p_{s}$ and saturation temperature $T_{s}$, $f\left(p_{c},T_{d/f}\right)$ is the enhancement factor of the mixture at dew/frost point temperature $T_{d}/T_{f}$ and pressure $p_{c}$ in the test chamber, $e_{w}\left(T_{s}\right)$ and $e_{w}\left(T_{d/f}\right)$ are the saturation water vapor pressure over a plane surface of the pure phase of liquid water or solid ice at the saturator temperature $T_{s}$ and dew/frost point temperature $T_{d}/T_{f}$, respectively.

The combined standard deviation $\sigma \left(T_{d/f}\right)$ of dew/frost point temperature is derived from Equations (6), (7) and (11). Thus:

(12) $$\sigma \left(T_{d/f}\right)=\sqrt{\sigma^{2}\left[e_{w}\left(T_{d/f}\right)\right]+\sigma^{2}\left[f\left(p_{c},T_{d/f}\right)\right]+\sigma^{2}\left[e_{w}\left(T_{s}\right)\right]+\left(\sigma^{2}\left(p_{c}\right)\right)+\sigma^{2}\left[f\left(p_{s},T_{s}\right)\right]+\left(\sigma^{2}\left(p_{s}\right)\right)}$$

A detailed description on uncertainty evaluation of the water vapor partial pressure is given in Equation (13).

(13) $$\sigma_{e_{w}(d/f)}=\sqrt{{\left(\frac{\partial e}{\partial A}\right)}^{2}\sigma_{A}^{2}+{\left(\frac{\partial e}{\partial S}\right)}^{2}\sigma_{S}^{2}+{\left(\frac{\partial e}{\partial L}\right)}^{2}\sigma_{L}^{2}}$$

where $\sigma_{A}{,}^{}\sigma_{S}{,}^{}\sigma_{L}$ is the standard deviation of integral absorption A, line strength S and optical pathlength L. According to Equation (5), we can calculate the partial differential in Equation (13), and since the temperature dependency of enhancement factors is very small, little error would be induced by temperatures while at low to moderate pressures (< 10 MPa). The maximum standard deviation is < 0.15% when dew/frost point temperature range is −100 °C ~ 0 °C [22]. Because the SNR is inconsistent in different absorbance, the fitting deviation of integral absorption $\sigma_{A}$ is not the same; it is the maximum when the absorption is minimal and is < 1.36% for 7242.37 cm^−1^ line, < 1.14% for 7243.07 cm^−1^ line. The uncertainty of water vapor partial pressure is < 1.41% for 7242.37 cm^−1^ line and < 1.18% for 7243.07 cm^−1^ line. The uncertainty of pressure is < 0.2%, so the maximum combined standard deviation of dew/frost point temperature is < 1.998%. Because the dew/frost point temperature range of the experimental measurement is −80 °C ~ 10 °C, this precision is only applicable to this range. For the range of −80 °C ~ −93 °C, the estimated accuracy is < ± 2% through the simulated absorbance.

## 5. Results and discussion

### 5.1. TDLAS dew/frost point temperature sensor detection limits analysis

**Figure 7:**
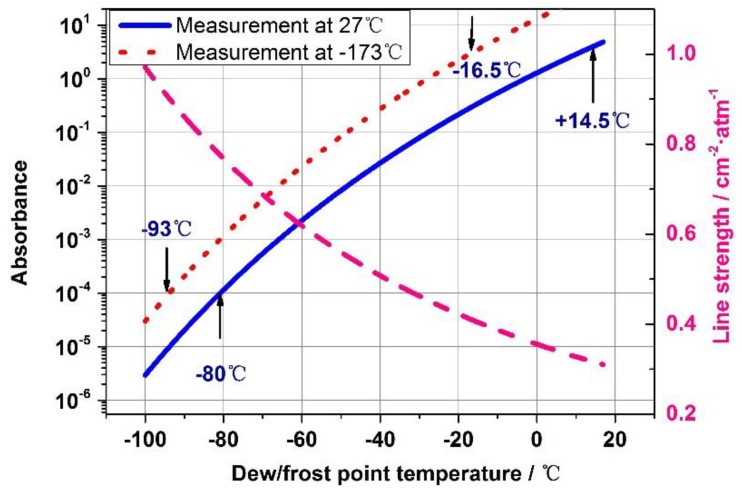
Simulation of the water vapor 7242.37 cm^−1^ and 7243.08 cm^−1^ peak absorption in air under different dew/frost point temperature conditions and a pressure of 1 atm. The red dotted line is the result of a gas temperature of −173 °C. In this case, the water vapor would be supersaturated. The blue solid line is the result of a gas temperature equals 27 °C. The line strength at different temperature (pink dash line) are based on the spectroscopic database HITRAN. (Note: the horizontal axis label for the line strength curve should simply read temperature not dew/frost point.).

The cryogenic wind tunnel could operate within a temperature range from −173 °C to +27 °C at a pressure of 1atm. The air from the cryogenic wind tunnel was extracted into the multipass absorption cell with a total 1150.2 cm optical pathlength for its water vapor contents measurements by the TDLAS technique. Simulated absorption of the 7242.37 cm^−1^ and 7243.08 cm^−1^ water vapor rotational-vibrational line at different dew point temperatures are shown in Figure 7.

For our TDLAS sensor’s nominal minimum detectable peak absorbance of 10^−4^, the corresponding minimum dew point temperature is about −93 °C assuming the measurements would be conducted at −173 °C (which was the lowest operation temperature of the cryogenic wind tunnel). In this case, the water vapor would be supersaturated. At a measurement temperature of 27 °C and a nominal maximum absorbance measurement of 4, the corresponding maximum dew point temperature is about 14.5 °C. The 7242.37 cm^−1^ and 7243.08 cm^−1^ water vapor rotational-vibrational line we used in this study has higher line strength at lower temperature. Line strength and absorption peak height is increase with gas temperature decrease, and so the overall measurement range of our TDLAS dew/frost point sensor is −93 °C ~ 14.5 °C when the TDLAS gas absorption measurements at operation temperature of the cryogenic wind tunnel from 27 °C to −173 °C.

### 5.2. Measurement result

**Figure 8:**
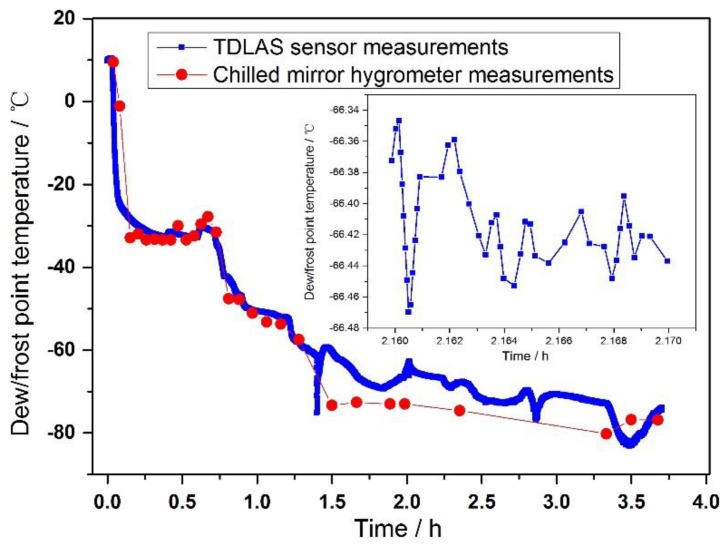
Dew point temperatures determined by both a TDLAS sensor and a chilled mirror hygrometer.

In the application for the determination of the dew/frost point temperature in a cryogenic wind tunnel, the water concentration in the extracted gas was determined by measuring the integrated absorbance of the same absorption lines. Together with the continuously recorded temperature and pressure of the TDLAS gas cell, the water vapor partial pressure and its corresponding dew/frost point temperature were then calculated by Equations (5)–(8). For comparison purposes, the dew/frost point temperature of the same gas flow was also measured simultaneously by a chilled mirror hygrometer. The measurement results are displayed in Figure 8.

As shown in Figure 8, the minimum dew point temperature in the cryogenic wind tunnel is about −82 °C. The agreement between our TDLAS dew/frost point results and the chilled mirror hygrometer was very good when dew point was higher than ~ −60 °C. However, when the dew point temperature was below ~ −60 °C, the TDLAS sensor results were about 5 °C higher than those of the chilled mirror hygrometer. This will be discussed in the next Section 5.3. Due to its operation principle, the chilled-mirror hygrometer took minutes to make a single measurement. In contrast, a significant advantage of the optical TDLAS technique is its rapid response time of less than a second. Therefore, the TDLAS dew/frost point temperature sensor will be suitable for fast-change environment of low dew temperature, such as cryogenic wind tunnels. Through detailed analysis, we determined the uncertainty of the dew/frost point temperature is < 2% of measurement results.

### 5.3. Deviation analysis

**Figure 9:**
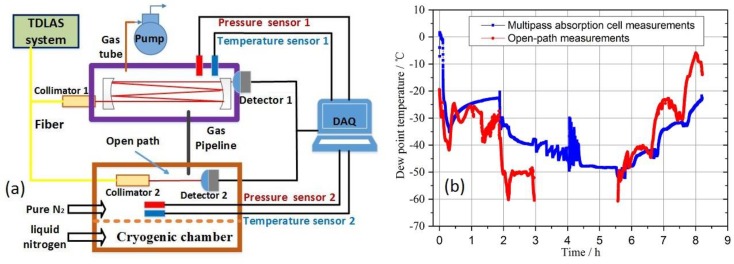
(**a**) The schematic diagram of a setup to verify impacts of gas pipeline absorption on water vapor concentration and dew point determination. DAQ: data acquisition and processing; (**b**) Results based on long multipass and short open-path absorption measurements. Note: during the very low water vapor concentration and dew point temperature time interval (3–5.5 h), the short open-path did not have sufficient sensitivity to make measurements.

For frost temperatures below ~ −60 °C as in Figure 8, the measurement results of TDLAS were about 5 °C higher than those of the chilled-mirror hygrometer. We propose that this was due to water vapor adsorptions in the gas handling pipeline. At low dew point situations, the concentration of water vapor is very low, any adsorption or release of water vapor to or from the wall/surface of gas handling pipes can impact on the final concentration at the point of measurements. In order to confirm this conjecture, a comparison experiment for simple qualitative analysis was performed. Figure 9a is a schematic diagram of the experiment.

As shown in Figure 9a, the multipass TDLAS sensor was connected to a cryogenic chamber via a gas handling pipeline. The temperature inside the cryogenic chamber could be varied from room temperature to −173 °C through liquid nitrogen refrigeration. The pressure inside the chamber was controlled slightly above the surrounding atmospheric air pressure by flowing in high purity nitrogen. An open-path setup was placed directly inside the cryogenic chamber for in-situ dew/frost point detection. Its optical path length is 400 cm. Air in the cryogenic chamber was flowed through the gas handling pipeline into the multipass absorption cell. The length of the gas pipeline was about 1400 cm. Laser output radiation from the DFB diode laser was divided into two parts by a 1 × 2 50:50 fiber splitter. One beam passed through the 400 cm open-path setup and was utilized for in-situ measurements of water vapor dew/frost point, whereas the other beam passed through the 1150.2 cm multipass absorption cell as in its normal configuration in Figure 1. After several hours cleaning with high purity nitrogen, two sets of devices conducted dew/frost point measurements for several hours simultaneously. Such measurements were repeated several times. A typical set of similar experimental results are shown in Figure 9b. Because the effective optical pathlength of the open-path device is only 400 cm, its frost point detection limit was estimated about −63 °C. Therefore, frost point temperatures below this detection limit were missing during the time interval (3–5.5 h) in the Figure 9b.

As shown in the Figure 9b, dew/frost point temperatures in the multipass absorption cell were higher than that in the cryogenic chamber in the process of cooling, and then became lower than that in cryogenic chamber in the process of heating up. This was because the water vapor concentration in the cryogenic chamber gradually decreases during the cooling process, so the water vapor on the gas pipeline and enclosure surface was continuously desorbed to ensure that the vapor concentration reaches equilibrium, which led to water vapor concentrations and dew/frost point temperatures in multipass absorption cell were higher than that in cryogenic chamber. Conversely, the water vapor concentration in the cryogenic chamber gradually rose during the heating process, so the water vapor was continuously absorbed onto the gas pipeline and enclosure surface to ensure that the vapor concentration reaches equilibrium, which led to dew/frost point temperatures in the multipass absorption cell were lower than that in the cryogenic chamber. Through this experiment, we demonstrated that the adsorption process of water vapor onto gas pipeline and other surfaces can affect the dew/frost point temperature and require some time to establish an equilibrium.

## 6. Conclusion and outlook

This research presented a laser dew/frost point temperature sensor based on absorption spectroscopy of water vapor. The sensor was validated in a blind comparison against a chilled-mirror hygrometer in a cryogenic wind tunnel application. During several measurement runs, the TDLAS dew/frost point sensor achieves a minimum frost point temperature of ~ −80 °C. An excellent agreement with the chilled-mirror hygrometer was achieved at higher dew/frost point temperatures (> −60 °C), but there were 5 °C deviations at lower dew point temperature (< −60 °C). This deviation was due to the surface water vapor absorption/release, leading to air that entering the chilled mirror hygrometer is drier. The time response of TDLAS dew/frost point sensor was about 0.8 s, but that of chilled mirror hygrometer was about several minutes, or even several hours at lower dew point conditions. Further advantages of the TDLAS-based sensor include non-intrusive and continuous measurements, and low dew/frost point temperature sensing. It is feasible to miniaturize and integrate such an optical sensor into application environments to make in-situ high accuracy measurements, e.g. for cryogenic wind tunnels and airborne atmospheric researches.

## Figures and Tables

**Table 1:** Brands and some parameters of several commercially available dew/frost point instruments ^a^.

Brand	Sensor type	Measurement range	Accuracy	Response time ^b^	Operation environment
GE (MMY30)	Aluminum oxide sensor	−90 ~ +10 °C	± 2 °C (25 °C)	-	−40 ~ +50 °C
PhyMetrix (DewPatrol)	Nanopore sensor	−110 °C ~ +20 °C	± 2 °C	3 min	−20 ~ +60 °C Non-corrosive
Michell (Easidew)	Ceramic moisture sensor	−100 °C ~ +20 °C	≤ ± 2 °C	5 min (dry to wet)	−40 ~ +60 °C Non-corrosive
Vaisala (DMT340)	Capacitive thin-film polymer sensor	−70 °C ~ +80 °C	< ± 3 °C	10 min (wet to dry)	−40 ~ +80 °C Non-corrosive
SIDPH (FM860)	Dual ceramic nano thin-film sensor	−110 °C ~ +20 °C	± 2 °C	1 min (dry to wet)	−40 ~ +65 °C Non-corrosive
MBW (373LX)	Chilled mirror hygrometry sensor	−95 °C ~ +20 °C	± 0.1 °C	~ 1 min	15 ~ +35 °C Non-corrosive

^a^ These data are from their manufacturer’s website.

^b^ Response time from dry to wet is shorter than wet to dry. In an extremely low dew/frost point environment, the response time is greater than the value in the list.

**Table 2:** H_2_O absorption line intensities.

Frequency $\nu_{0}$ (cm^−1^)	Lower state energy $E^{\prime \prime }$ (cm^−1^)	Line intensity S @296 K (cm^−1^/ (molecule·cm^−2^))		Uncertainty of intensity	
		HITRAN 2012	Our work	HITRAN 2012	Our work
7242.37075	42.3717	1.19 × 10^−20^	1.188 × 10^−20^	≥ 5% and ≤ 10%	1.023%
7243.07526	134.9016	1.61 × 10^−20^	1.626 × 10^−20^	≥ 5% and ≤ 10%	1.312%
